# Quality control and quantification in IG/TR next-generation sequencing marker identification: protocols and bioinformatic functionalities by EuroClonality-NGS

**DOI:** 10.1038/s41375-019-0499-4

**Published:** 2019-06-21

**Authors:** Henrik Knecht, Tomas Reigl, Michaela Kotrová, Franziska Appelt, Peter Stewart, Vojtech Bystry, Adam Krejci, Andrea Grioni, Karol Pal, Kamila Stranska, Karla Plevova, Jos Rijntjes, Simona Songia, Michael Svatoň, Eva Froňková, Jack Bartram, Blanca Scheijen, Dietrich Herrmann, Ramón García-Sanz, Jeremy Hancock, John Moppett, Jacques J. M. van Dongen, Giovanni Cazzaniga, Frédéric Davi, Patricia J. T. A. Groenen, Michael Hummel, Elizabeth A. Macintyre, Kostas Stamatopoulos, Jan Trka, Anton W. Langerak, David Gonzalez, Christiane Pott, Monika Brüggemann, Nikos Darzentas, on behalf of the EuroClonality-NGS Working Group

**Affiliations:** 1 Department of Hematology, University Hospital Schleswig-Holstein, Kiel, Germany; 2 Central European Institute of Technology, Masaryk University, Brno, Czech Republic; 3 Centre for Cancer Research and Cell Biology, Queen’s University Belfast, Belfast, UK; 4 Centro Ricerca Tettamanti, University of Milano Bicocca, Monza, Italy; 5 Department of Internal Medicine – Hematology and Oncology, University Hospital Brno and Faculty of Medicine, Masaryk University, Brno, Czech Republic; 6 Department of Pathology, Radboud University Medical Center, Nijmegen, The Netherlands; 7 CLIP – Childhood Leukaemia Investigation Prague, Department of Paediatric Haematology and Oncology, Second Faculty of Medicine, Charles University, University Hospital Motol, Prague, Czech Republic; 8 Department of Paediatric Haematology, Great Ormond Street Hospital, London, UK; 9 IBMCC-CSIC, Hospital Universitario de Salamanca-IBSAL, Salamanca, Spain; 10 Bristol Genetics Laboratory, Southmead Hospital, Bristol, UK; 11 Department of Pediatric Haematology, Bristol Royal Hospital for Children, Bristol, UK; 12 Department of Immunohematology and Blood Transfusion (IHB), Leiden University Medical Center, Leiden, The Netherlands; 13 Department of Hematology, Hopital Pitié-Salpêtrière, Paris, France; 14 Insititute of Pathology, Charité – Universitätsmedizin Berlin, Berlin, Germany; 15 Department of Hematology, APHP Necker-Enfants Malades and Paris Descartes University, Paris, France; 16 Institute of Applied Biosciences, Centre for Research and Technology Hellas, Thessaloniki, Greece; 17 Department of Immunology, Laboratory Medical Immunology, Erasmus MC, University Medical Center, Rotterdam, The Netherlands

**Keywords:** Genetics research, Cancer genetics

## Abstract

Assessment of clonality, marker identification and measurement of minimal residual disease (MRD) of immunoglobulin (IG) and T cell receptor (TR) gene rearrangements in lymphoid neoplasms using next-generation sequencing (NGS) is currently under intensive development for use in clinical diagnostics. So far, however, there is a lack of suitable quality control (QC) options with regard to standardisation and quality metrics to ensure robust clinical application of such approaches. The EuroClonality-NGS Working Group has therefore established two types of QCs to accompany the NGS-based IG/TR assays. First, a central polytarget QC (cPT-QC) is used to monitor the primer performance of each of the EuroClonality multiplex NGS assays; second, a standardised human cell line-based DNA control is spiked into each patient DNA sample to work as a central in-tube QC and calibrator for MRD quantification (cIT-QC). Having integrated those two reference standards in the ARResT/Interrogate bioinformatic platform, EuroClonality-NGS provides a complete protocol for standardised IG/TR gene rearrangement analysis by NGS with high reproducibility, accuracy and precision for valid marker identification and quantification in diagnostics of lymphoid malignancies.

## Introduction

Identification and assessment of clonal immunoglobulin (IG) and T cell receptor (TR) gene rearrangements is a widely used tool for the diagnosis of lymphoid malignancies, and is also essential for monitoring minimal residual disease (MRD) [1–6].

Next-generation sequencing (NGS) of IG/TR gene rearrangements is gaining popularity in clinical laboratories, as it avoids laborious design of patient-specific real-time quantitative (RQ)-PCR assays and provides the capability to sequence multiple rearrangements and rearrangement types within a single sequencing run. It also allows detection of MRD with a more specific readout than RQ-PCR [7]. Hence, several methods have already been described for high-throughput profiling of IG/TR rearrangements at diagnosis and follow-up in acute lymphoblastic leukaemia (ALL), chronic lymphocytic leukaemia (CLL) and other lymphoid malignancies [8–13].

NGS assays, especially those based on amplicons, pose major challenges, as multiple primers need to anneal under the same reaction conditions, while many technical variables may be introduced by library preparation, sequencing and bioinformatics, potentially leading to inaccurate results [14]. Particularly in a clinical context, strategies for standardisation of laboratory protocols and quality control (QC) of each component of an NGS assay are highly desirable, if not required.

Reference standards are essential for the evaluation of wet-lab and in silico NGS processes to ensure the analytical validity of test results prior to implementation of an NGS technology into clinical practice [15–17]. Reference DNA materials should be stable sources of rearrangements that can be sequenced and used for measuring qualitative and quantitative properties. However, previously published standards have a limited scope and utility, since they (1) do not cover all relevant IG/TR loci, (2) do not report on the quality of the sequencing run or the performance of samples and primers and/or (3) are synthetic constructs that may not reflect the complexity of native genomic DNA [9, 18, 19].

The EuroClonality-NGS Working Group was initiated to develop, standardise and validate protocols for IG/TR NGS applications, as introduced in Langerak et al. [20] and described in the accompanying manuscripts by Brüggemann et al. [21] and Scheijen et al. [22]. Innovatively, the EuroClonality-NGS assays include two types of QCs, both based on basic assay components, and both fully integrated in ARResT/Interrogate [23], the interactive bioinformatics platform developed within the Working Group:

1. A central polytarget QC (cPT-QC) consisting of a standardised mixture of lymphoid specimens, representing a full repertoire of IG/TR genes. It serves to assess performance biases or unusual amplification shifts in a sequencing run by tracking primer usage and comparison with stored reference profiles.
2. A central in-tube quality/quantification control (cIT-QC) consisting of human B and T cell lines with well-defined IG/TR rearrangements. The cIT-QC is directly added to a sample to undergo concurrent library preparation and sequencing, acting as in-tube qualitative and quantitative standard that is subjected to the same technical downstream variables.

**Fig. 1:**
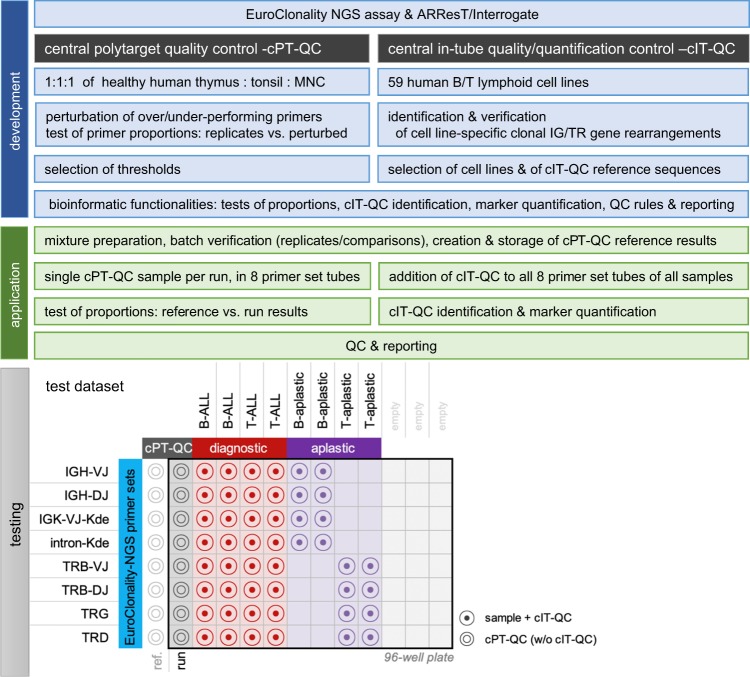
Study design: components and steps of development (in blue), application (in green) and testing for the central polytarget quality control (cPT-QC) and central in-tube quality/quantification control (cIT-QC), including a schematic overview of the test dataset based on a 96-well plate. Text boxes are either shared across cPT-QC and cIT-QC or describing equivalent steps if on same row. MNC = mononuclear cells, QC = quality control, ref. = reference, w/o = without

Here we describe, evaluate and showcase these concepts and functionalities. We tested the developed protocol on a dataset of polyclonal samples, B-ALL and T-ALL diagnostic materials and follow-ups of patients with substantial treatment-induced shifts in IG/TR repertoires. We show its successful application and robustness for clinical laboratories that want to implement the EuroClonality-NGS assays for marker identification and quantification. Figure 1 provides an overview of the study.

## Materials and methods

### EuroClonality-NGS assay

The EuroClonality-NGS assay for marker identification used herein is the two-step PCR protocol with eight primer sets (IGH-VJ, IGH-DJ, IGK-VJ-Kde, intron-Kde, TRB-VJ, TRB-DJ, TRG, TRD)—hereafter termed ‘tubes’—per sample, as described in the accompanying manuscript by Brüggemann et al. [21].

### ARResT/Interrogate

ARResT/Interrogate uses a web browser-based interface to (1) run an analytical pipeline to identify different types of rearrangements—‘junction classes’—across all IG/TR loci (Supplementary Table S1), (2) store, retrieve and report on runs, (3) allow highly varied analyses and visualisations and (4) enable purpose-built meta-analyses and applications. Bioinformatic analyses were performed with ARResT/Interrogate and purpose-built tools unless otherwise stated. Further implementation details are provided below and as Supplementary Information. The platform is currently freely available at arrest.tools/interrogate, hosted at the MetaCentrum and CERIT-SC centres in the Czech Republic.

### Implementation of the cPT-QC

#### Sources and methods

**Fig. 2:**
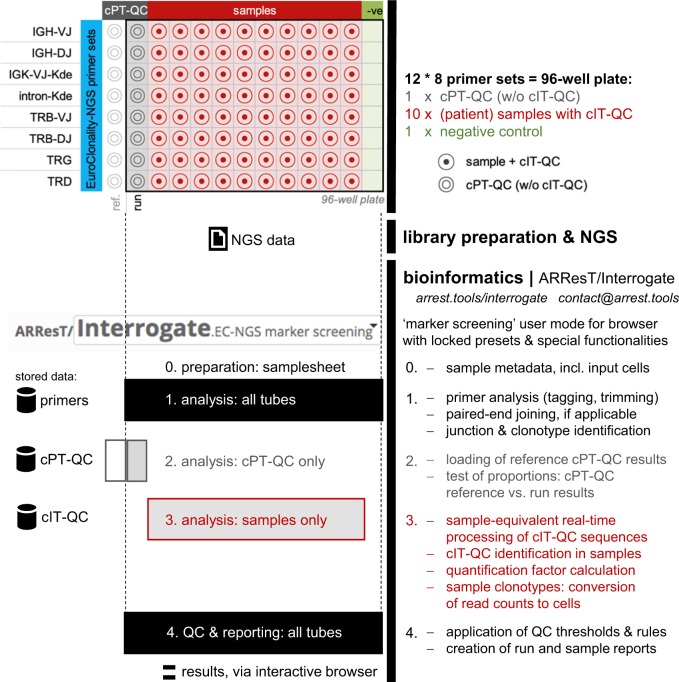
EuroClonality-NGS (next-generation sequencing) protocol for quality control and quantification in marker identification: 96-well plate set-up, including central polytarget quality control (cPT-QC) and central in-tube quality/quantification control (cIT-QC), library preparation and NGS, bioinformatics with ARResT/Interrogate. The bioinformatics are additionally organised per sample type to showcase distinct steps and functionalities listed on the right: all tubes (1 and 4, in black), cPT-QC (2, in grey), (patient) samples (3, in red)—these colours are shared with the well plate. ref. = reference, QC = quality control, w/o = without

The cPT-QC consists of genomic DNA isolated from healthy human thymus, tonsil and peripheral blood mononuclear cells (MNCs) in a 1:1:1 ratio (see Supplementary Information). The cPT-QC undergoes library preparation alongside the investigated samples (Figs. 1 and 2).

#### Implementation

Primers are bioinformatically identified in the reads of each of the eight cPT-QC tubes of the run and their abundances compared to stored cPT-QC reference results using the test of proportions.

Stored reference results are the output of ARResT/Interrogate from the analysis of a cPT-QC sample. These results should be confirmed through replicate runs over time in each lab to accommodate for technical variability (see Discussion). The results (and not the raw NGS data) are stored to ensure that the bioinformatic analysis is not compromised inadvertently by the user; this means that the results are updated with every major release of ARResT/Interrogate to ensure compatibility with new runs.

Issues with abundances of primers of a specific primer set are used to tag the corresponding cPT-QC samples and all user samples of the same primer set as ‘QC-failed’.

#### Replicates

As reproducibility is important for a QC of this type, we performed replicate runs of cPT-QC and also of MNC (four libraries in total); MNCs are regularly used and could serve as an alternative. Relative abundances of 5′ primers were compared employing the test of proportions.

#### Primer perturbations

To investigate whether and how the cPT-QC can be used to detect issues with primer performance, artificial perturbations of primer concentrations were created to simulate missing pipetting a primer or pipetting the wrong primer concentration.

First, 5′ primer usage was analysed in a cPT-QC sample. Two primers of differing abundances were selected from each primer set, skipping intron-Kde that only has two primers: IGH-VJ-FR1-M-1, IGHV-FR1-O-1; IGHD-B-1, IGHD-E-1; IGK-V-G-1, IGK-V-I-1; TRB-V-AD-1, TRB-V-G-1; TRB-D-A-1, TRB-D-B-1; TRG-V-F-1, TRG-V-E-1; TRD-D-A-1, TRD-V-B-1. Second, these primers were perturbed by fully excluding them from the primer pool (0%) and by changing their concentration by reduction to 10% and by increase to 200%. Replicate runs of these three primer-perturbed cPT-QC libraries (six in total) were performed; however, since the replicates were consistent (data not shown), only the first replicate of each is shown in Results. Finally, relative abundances of 5′ primers were compared between normal replicates and between normal replicates and the perturbed libraries using the test of proportions.

### Design and validation of the cIT-QC

#### Sources and methods

In total, 59 human B (*n* = 30) and T (*n* = 29) lymphoid cell lines were obtained from the American Type Culture Collection (ATCC, Manassas, VA, USA; www.lgcpromochem-atcc.com) and the German Collection of Microorganisms and Cell Cultures GmbH (DSMZ, Braunschweig, Germany; www.dsmz.de), or were derived from internal cell line banks. Supplementary Table S2 gives an overview of the cell lines. DNA from cultured cell lines was isolated using a phenol–chloroform extraction protocol, followed by ethanol precipitation and elution in Tris ethylenediaminetetra-acetic acid buffer. Alternatively, DNA was isolated with the GenElute Mammalian Genomic DNA Miniprep Kit (Sigma-Aldrich, St. Louis, MO, USA) according to the manufacturer’s protocol.

#### Identification of cell line-specific clonal IG/TR gene rearrangements

Each of the 59 cell lines was screened for clonal IG/TR gene rearrangements using the EuroClonality-NGS assay with 100 ng of DNA (quantified with Qubit 3.0, Thermo Fisher Scientific) from each cell line, without the addition of MNC. Paired-end sequencing (2 × 250 bp) was performed on Illumina MiSeq (Illumina, San Diego, CA, USA) with a final concentration of 7 pM per library aiming for at least 2000 reads per sample. To avoid low-complexity issues, 10% PhiX control was added to each sequencing run.

#### Verification of cell line-specific clonal IG/TR gene rearrangements

Additional methods were used to verify the NGS amplicon-identified cell line rearrangements:

1. A capture-based protocol, established within EuroClonality-NGS Working Group and covering the coding V, D and J genes of IG/TR loci [13]: in short, cell line DNA was fragmented and processed with the KAPA Hyperplus Kit with Library Amplification (Roche Sequencing Solutions, Pleasanton, CA, USA); hybridisation of libraries was performed with customised SeqCap EZ Choice Probes (Roche Sequencing Solutions, Pleasanton, CA, USA), developed based on Wren et al. [13] 2  × 150 bp paired-end sequencing was performed on Illumina NextSeq.
2. Multiplex amplification and Sanger sequencing according to the BIOMED-2 protocol: PCR products were checked for fragment sizes and clonality in the QIAXCEL Advanced System [24, 25]. Clonal PCR products were subjected to heteroduplex analysis and sequenced on either an ABI 3130 or ABI 3500 platform (Applied Biosystems, Foster City, CA, USA).

IG/TR rearrangement profiles of all cell lines were compared between the different methods.

For cases with discrepant results between the three methods, IG/TR allele-specific PCR assays were designed for digital droplet PCR (ddPCR) (QX200TM Droplet DigitalTM PCR System, Bio-Rad) to verify the respective rearrangement. Absolute quantification of IG/TR gene rearrangements by ddPCR was performed using two different genomic DNA amounts (50 ng, 100 ng) (Supplementary Information). Each experiment included a polyclonal MNC control and a no-template control.

#### Cell line selection criteria

For establishment of the cIT-QC from the spectrum of IG/TR gene rearrangements of the 59 cell lines, the following selection criteria were defined:

1. The final set should consist of as few cell lines as possible, while covering each primer set by at least three different rearrangements, hence aiming for ALL cell lines harbouring not only lineage characteristic but also cross-lineage rearrangements.
2. The rearrangements should be unambiguously detectable with Sanger sequencing and amplicon-based NGS.
3. The variable region of IGHV-(IGHD)-IGHJ gene rearrangements should preferably be unmutated in order to avoid issues with primer annealing.

#### Implementation

For cIT-QC mixture preparation see Supplementary Information.

Bioinformatically, cIT-QC reads are identified using an immunogenetic annotation-based approach that is extremely fast while allowing for variations in sequence, avoiding compute-intensive and potentially inaccurate alignment.

For QC, we expect identification of at least one read per cIT-QC rearrangement and of at least as many total cIT-QC reads as total cIT-QC cells, otherwise the tube is tagged as ‘QC-failed’ (see below for how this is used in ARResT/Interrogate).

Quantification applies the quantification factor—calculated per primer set by dividing total cIT-QC cells by total cIT-QC reads—to convert read counts of a clonotype to cell counts, and then calculate its relative abundance against the total sample input cells.

### Creation of a test dataset

To evaluate and showcase the aforementioned concepts and functionalities, we compiled a test dataset with:

1. Four diagnostic bone marrow B-/T-ALL samples with high leukaemic infiltration (assessed by routine cytomorphology to be 60–80%).
2. Four samples of patients with B/T cell aplasia after antibody treatment. The two samples with B cell aplasia were CLL samples after Rituximab (anti-CD20) treatment and the two samples with T cell aplasia were T cell prolymphocytic leukaemia samples after Alemtuzumab (anti-CD52) treatment. In all these samples lineage-specific aplasia was confirmed by flow cytometry.
3. cPT-QC for all primer sets, but with the TRB-VJ primer set results swapped with perturbed results from experiments outlined above. To showcase generic QC functionalities, one diagnostic sample was subsampled to <1000 random reads.

The diagnostic samples and the cPT-QC were run with all primer sets as described in the accompanying manuscript by Brüggemann et al. [21], while the aplastic follow-up samples only with the corresponding primer sets, that is, the IG sets for samples with B cell aplasia, and the TR sets for samples with T cell aplasia. Figure 1 includes a schematic of the test dataset. Finally, the follow-up samples were run without the addition of MNC to test that the addition of cIT-QC is sufficient to stabilise the samples for sequencing without compromising their immunogenetic profile.

## Results

The resulting protocol and functionalities for QC and quantification in IG/TR NGS marker identification are depicted in Fig. 2. We present and further discuss the underlying results below.

### cPT-QC allows to assess primer performance

We compared normal cPT-QC and MNC replicate libraries and primer-perturbed cPT-QC replicate libraries (10 libraries in total) to investigate the use of cPT-QC in assessing primer performance. We applied the test of proportions on 5′ primer relative abundances in those libraries, which showed that there is a clear difference in *p* values between un-perturbed (high *p* values indicating insignificant changes) and perturbed (low *p* values) primers. In other words, *p* values of the differences in abundance of the perturbed primers are noticeably lower, an observation we can use to highlight such cases.

Table 1 presents a simplified view of the results, focusing on perturbed primers plus at least one other un-perturbed primer per primer set, either to show their normal behaviour or discuss their abnormal behaviour. At a *p* value threshold of 1e^−200^ none of the primers are flagged in the cPT-QC (white cells), which highlights the reproducibility of the assay, while all the perturbed primers are flagged in the perturbed libraries (light/dark grey cells). Significant changes in abundance are also visible in other cells, with the most likely explanation that those primers were indirectly affected by perturbations of other primers. That is, a primer ‘taking over’ when an initially abundant primer was excluded, such as IGHV-FR1-D-1 when IGH-VJ-FR1-M-1 is perturbed either way, especially since these primers amplify partially overlapping lists of genes. Supplementary Table S3 presents the full set of results, including the actual *p* values and results from the replicate MNC libraries.

**Table 1:**
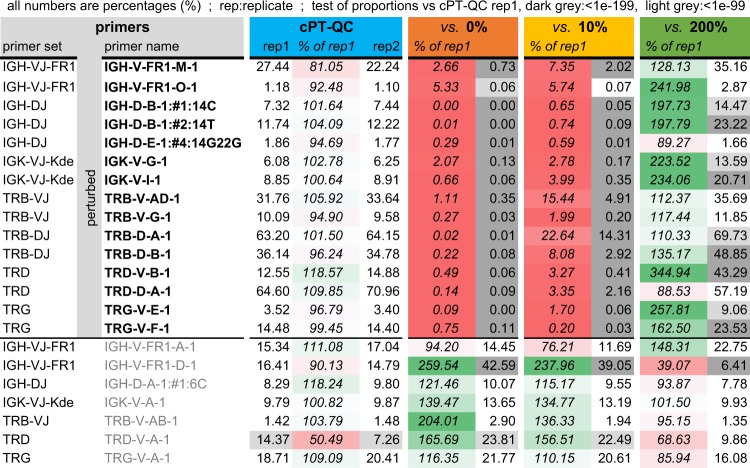
cPT-QC: replicates and primer perturbations. Relative abundances (%) of selected 5′ primers across all primer sets. Top group of primers were perturbed as described in Materials and methods; bottom group is a selection of primers that were left un-perturbed: one per primer set selected alphabetically, plus two examples where the primer behaviour is of interest to the discussion (see text). Results are shown from two cPT-QC replicates (blue column) and from replicate 1 of the blue column (“rep1”) vs. cPT-QC libraries where primers were excluded (0%, orange column), reduced to 10% (yellow column) and increased to 200% (green column). Changes in abundance compared to cPT-QC rep1 are shown separately (column “% or rep1”, in italics) and coloured from red (0%) to white (100%, i.e. no change) to green (200%). Actual primer abundances are coloured based on the *p* value from the test of proportions, with grey indicating a noticeable change according to our threshold of 1e−200 (*p* value <1e−199 highlighted in dark grey, and <1e−99 in light grey, otherwise in white)

### Composing the cIT-QC sample from human B and T cell lines

Following the criteria outlined above, we selected six B cell lines: ALL/MIK (ALL), Raji (Burkitt lymphoma), REH (B cell precursor ALL), TMM (CML-BC/EBV + B-LCL), TOM-1 (ALL) and WSU-NHL (B cell lymphoma, histiocytic lymphoma); and three T cell lines: JB6 (ALCL), Karpas299 (ALCL) and MOLT-13 (ALL). The nine cell lines featured a total of 46 rearrangements, all of which are used as part of the cIT-QC. All but two rearrangements that were not detected by capture NGS were detected by all three sequencing methods. Also, another two were of very low abundance and/or trimmed in the capture NGS data, but since the junction segmentation was clearly the same, they were still tagged as confirmed. Table 2 presents the full list of the 46 rearrangements, with the NGS amplicon-based reference nucleotide sequences in Supplementary Table S4.

**Table 2:** cIT-QC: full list of gene rearrangements per primer set and human B/T cell line, with notes on their verification and clonotype annotation

Primer set	Cell line	Notes	Clonotype (see Supplementary Information—Materials and methods)
TRB-VJ	JB6		VJ:Vb-(Db)-Jb V12–3 = V12–4 6/14/4 J2–3 CASRLAGGPDTQYF pro
TRB-DJ	JB6		DJ:Db-Jb D1 7/6/4 J2–2 VGTEITGELFF pop
TRG	JB6		VJ:Vg-Jg V10 7/12/12 J1 = J2 CAAWS*GW#KLF unp
TRG	JB6		VJ:Vg-Jg V2 5/13/ J1 = J2 CATWGSI*VNYYKKLF unp
TRB-VJ	Karpas299		VJ:Vb-(Db)-Jb V20–1 1/22/6 J2–7 CSARAQIGSSPLEQYF pro
TRB-DJ	Karpas299		DJ:Db-Jb D1 /2/6 J1–6 VGTGGLNSPLHF pop
TRG	Karpas299		VJ:Vg-Jg V2 /13/4 JP2 CATWDGG*VP#SDWIKTF unp
TRG	Karpas299		VJ:Vg-Jg V8 /2/5 J1 = J2 CATWDR##YKKLF unp
IGH-VJ-FR1	ALL/MIK		VJ:Vh-(Dh)-Jh V3–72 16/24/ J4 SPCPPRKN#YFDYW unp
IGH-VJ-FR1	ALL/MIK		VJ:Vh-(Dh)-Jh V7–4–1 11/40/27 J4 TPYYYDSSGY*VP unp
IGK-VJ-Kde	ALL/MIK		Vk-Kde V2–24 = V2D-24 26/6/20 Kde LGGR unk
IGK-VJ-Kde	ALL/MIK		VJ:Vk-Jk V1–39 = V1D-39 6/7/5 J3 CQQSYSTGA#F unp
intron-Kde	ALL/MIK		Intron-Kde intron 4/2/ Kde PCVCPIDAAVASFP##SPSGSPGR unk
Intron-Kde	ALL/MIK	Capture: low%	Intron-Kde intron 4/6/1 Kde PCVCPIDAAVASFPSL#SPSGSPGR unk
TRD	ALL/MIK		VJ:Vd-(Dd)-Ja V2 5/21/4 J29 CACAQGGPRS#SGNTPLVF unp
TRG	ALL/MIK		VJ:Vg-Jg V2 /5/8 JP1 CATWDGP#GWFKIF unp
TRG	ALL/MIK		VJ:Vg-Jg V5 2/3/ JP1 CATWDTYTTGWFKIF pro
TRB-VJ	MOLT-13		VJ:Vb-(Db)-Jb V10–1 6/18/1 J1–1 CASRRVRRDRNTEAFF unp
TRB-DJ	MOLT-13		DJ:Db-Jb D1 //6 J1–5 VGTGG#QPQHF pop
TRB-DJ	MOLT-13		DJ:Db-Jb D2 /4/3 J2–3 VGTSGRA#TDTQYF pop
TRD	MOLT-13		VJ:Vd-(Dd)-Jd V1 1/9/ J1 CALGEPGGYTDKLIF pro
TRG	MOLT-13		VJ:Vg-Jg V3 /8/9 J1 = J2 CATWDRPRLKKLF pro
TRG	MOLT-13		VJ:Vg-Jg V8 3//3 JP1 CATWD#TGWFKIF unp
IGH-VJ-FR1	Raji	Capture: low%	VJ:Vh-(Dh)-Jh V3–11 = V3–21 = V3–48 2/40/3 J4 CARQRNDFSDNNSYYSNFDFW pro
IGH-DJ	Raji		DJ:Dh-Jh D6–13 8/12/6 J1 VGYSSIPPP#YFQHW pop
IGK-VJ-Kde	Raji		Vk-Kde V1–8 2/2/4 Kde CQQYYSYSVPSGSPGR unk
IGH-VJ-FR1	REH		VJ:Vh-(Dh)-Jh V3–15 1/21/5 J6 CTTGMVRGVI#YYYYGMDVW unp
IGK-VJ-Kde	REH		VJ:Vk-Jk V2–29 5/4/ J4 *MQGIHLS#LTF unp
IGK-VJ-Kde	REH		Vk-Kde V3–20 = V3D-20 4/1/ Kde CQQYGSS##SPSGSPGR unk
Intron-Kde	REH		Intron-Kde intron 5// Kde PCVCPINAAVASF##SPSGSPGR unk
TRB-VJ	REH		VJ:Vb-(Db)-Jb V20–1 1/2/26 J2–7 CSARG unp
TRD	REH		VD:Vd-Dd3 V2 7/3/ D3 CACLLGDTH unk
TRD	REH		VJ:Vd-(Dd)-Ja V2 3/22/5 J29 CACDPYGGGSP#SGNTPLVF unp
TRG	REH		VJ:Vg-Jg V9 1/2/3 J1 = J2 CALWEV#YYKKLF unp
TRG	REH		VJ:Vg-Jg V4 10/14/3 J1 = J2 CATLF*R#YYKKLF unp
IGH-VJ-FR1	TMM		VJ:Vh-(Dh)-Jh V1–24 /28/8 J5 CATDQAISGVVKSFDPW pro
IGH-DJ	TMM		DJ:Dh-Jh D2–2 3/13/ J3 VRIL**YQLLLNSANDAFDIW pop
IGK-VJ-Kde	TMM		Vk-Kde V2–30 = V2D-30 /7/3 Kde CMQGTHWRPGR#PSGSPGR unk
IGH-VJ-FR1	TOM-1		VJ:Vh-(Dh)-Jh V4–55 1/17/10 J6 CARWAGTTG#YYGMDVW unp
TRD	TOM-1		VD:Vd-Dd3 V2 3/3/2 D3 CACDL#GDTH unk
TRD	TOM-1		VD:Vd-Dd3 V2 8/4/ D3 CAFLLGDTH unk
TRG	TOM-1		VJ:Vg-Jg V5 8//18 J1 = J2 CAT#F unp
IGH-VJ-FR1	WSU-NHL		VJ:Vh-(Dh)-Jh V6–1 1/22/19 J6 CARGTYAAKASMDVW pro
IGH-DJ	WSU-NHL		DJ:Dh-Jh D2–2 1/1/8 J4 VRIL**YQLLY#DYW pop
IGK-VJ-Kde	WSU-NHL	Not in capture	VJ:Vk-Jk V1–17 = V1D-17 1//4 J4 CLQHNSYP#TF unp
Intron-Kde	WSU-NHL	Not in capture	Intron-Kde intron 2//3 Kde PCVCPIDAAVASFP##PSGSPGR unk

See Supplementary Table S4 for NGS amplicon-based full nucleotide reference sequences. *cIT-QC* central in-tube quality/quantification control

### QC aspects can be evaluated in ARResT/Interrogate

Information on the in silico QC based on both the cPT-QC and cIT-QC is available in ARResT/Interrogate (Supplementary Figure S1). Generic QC is also performed on samples, specifically to check for low number of raw reads and low percentage of reads with an identified junction. Such samples are tagged as ‘QC-failed’ and excluded by default to prevent the user from their unintended use. However, the user is notified and has the option to include them back in the analysis.

### Marker identification and quantification

Abundances of lymphocyte subpopulations are frequently not available for samples of patients with lymphoid malignancies. Furthermore, as IG/TR NGS only reflects relative representation of the rearrangements, it was important to establish a calibrator that would allow us to normalise sequencing reads to input DNA cells.

**Fig. 3:**
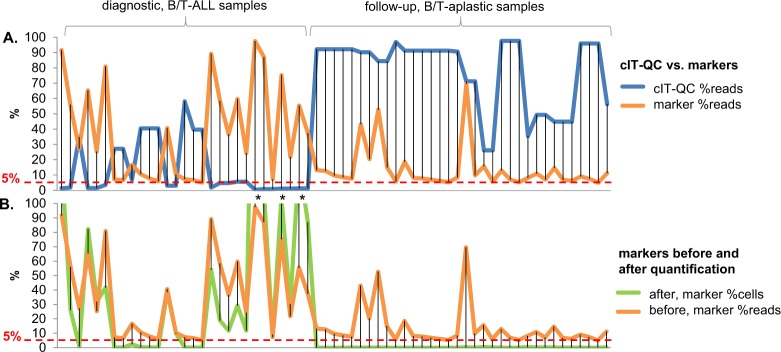
Abundances of central in-tube quality/quantification control (cIT-QC) and of markers before and after quantification, in the test dataset. The line of marker abundances before quantification (in orange) is shared in both plots for reference. The 5% threshold used for marker identification is shown in both plots. **a** Abundance in percentage of reads (“%reads”) of cIT-QC (in blue) and of markers before quantification (in orange), in diagnostic (left half) and follow-up aplastic (right half) samples. As expected because of the nature of the samples, the cIT-QC is generally most abundant where patient cells with clonal rearrangements are not, and vice versa. Note: For cIT-QC (in blue), the denominator is all reads with junction; for markers (in orange), it is what we term ‘usable’ reads with junction, which excludes cIT-QC reads; this may lead to sums of those two numbers that exceed 100% per sample. **b** Abundance of markers before (in orange) and after (in green) cIT-QC-based quantification to percentage of patient input cells (“%cells”). Quantification of markers in the aplastic samples places their abundances below the 5% threshold routinely used in marker identification and in the EuroClonality-NGS protocols. Note: When cIT-QC read counts are very low, indicating clonality, quantification factors may lead %cells to exceed 100%; three such cases in the test dataset are indicated by an asterisk (“ * ”)

Analysis of our test dataset showed the utility of the cIT-QC in marker identification and quantification. Excluding cIT-QC reads, both diagnostic and aplastic samples seem to harbour few highly abundant clones if simply based on the number of reads (Fig. 3, Supplementary Table S5). However, the very high number of reads from only a very limited number of cIT-QC cells (120–440, dependent on the number of cIT-QC rearrangements per primer set), in all aplastic and a few of the diagnostic samples, are an indirect yet clear indication of the restricted numbers of patient cells harbouring rearrangements in those samples. From another perspective, the total percentage of reads of cIT-QC is much greater than that of patient rearrangements in those samples, suggesting that also cIT-QC cells are more numerous than patient cells with rearrangements. Consequently, after quantification with the cIT-QC, marker abundances fall well below the threshold indicating clonality. On the other hand, and as expected, in most diagnostic samples cIT-QC reads constitute a minority, indicating the true abundant presence of patient cells with clonal rearrangements. Hence, using the cIT-QC, a marker can be more accurately quantified and identified.

### ARResT/Interrogate user mode for marker identification

A critical aspect of bioinformatic-based protocols is their standardisation and usability, as evident from our experiences within EuroClonality-NGS and EuroMRD. We have thus designed ARResT/Interrogate to be flexible but also ‘lockable’. Flexibility comes from a deep parameterisation of many aspects of the pipeline and the browser. At the same time, we can lock down important parameters so that users cannot inadvertently compromise the analysis. This concept is called ‘user mode’ in ARResT/Interrogate, and as a result of this study we have created a marker identification user mode.

In this user mode, EuroClonality-NGS primer sets and cIT-QC sequences are pre-selected and locked, as are other pipeline options. A special samplesheet is available to annotate samples with metadata, including providing numbers of sample input cells for quantification. The user interface is simplified, with many non-essential functionalities (including many of the visualisations normally available) hidden from view, and with less user actions required to load results. The minimum read-based percentage abundance for a clonotype is pre-set to 5% for marker identification.

## Discussion

In this study, we introduce protocols developed within the EuroClonality-NGS Working Group for QC and quantification in NGS-based IG/TR marker identification. Both laboratory and bioinformatic protocols are presented and showcased on clinically relevant data.

The cPT-QC is used to monitor the primer performance of each of the EuroClonality multiplex NGS assays; the cIT-QC is spiked into each patient DNA sample for QC and quantification. The use of ‘central’ highlights that these controls should be as stable as possible and thus centrally available at an applicable level (minimum at an intra-laboratory level)—this is further discussed below in the context of the cPT-QC.

Our experiments show that the cPT-QC is a valuable tool to monitor reproducibility of results and to identify primer perturbations and other deviations in the wet-lab protocol, as they introduce detectable changes to the sequencing profile. The addition of cPT-QC to each analysis allows to check the primer and assay performance after sequencing. Accidental deviations in the concentrations of single primers within the multiplexed IG/TR primer sets can be detected, performance failures of single primers can be traced and consequences for the IG/TR analysis can be estimated by analysis of cPT-QC data.

In our study, replicates of cPT-QC demonstrated high reproducibility. Nevertheless, we are aware that reproducibility across labs may be affected by a large number of other variables, from consumables and equipment to users. Only centralised access to consumables, for example, in the form of a kit, and a comprehensive protocol, including the equipment used, will further improve inter-laboratory comparability of results. Besides, activities such as the QC rounds organised bi-annually by ESLHO (eslho.org) are an opportunity to gather data and experience, compare assay performance and identify relevant factors introducing variations. Until full inter-laboratory standardisation is guaranteed, the implementation of the cPT-QC will require that the reference samples are analysed in each laboratory separately, and updated with every new batch of reagents, while keeping track of equipment and users. These reference data can then be stored in ARResT/Interrogate, which has the ability to store as many different such sets of reference data as needed, for example, linking a specific set to a specific user if necessary.

In this study we also highlighted a number of unique and advantageous properties of the cIT-QC. In contrast to plasmids or synthetic reference templates, cIT-QC cell lines are particularly well suited to be used as control because they are sources of large quantities of genomic DNA. Second, the nine cell lines with a total of 46 rearrangements represent as few cell lines as possible while covering each primer set by at least three different rearrangements, taking advantage of ALL cell lines harbouring not only lineage-associated but also cross-lineage rearrangements. Third, the rearrangements are unambiguously detectable with amplicon-based NGS. Fourth, the variable region of IGHV-(IGHD)-IGHJ gene rearrangements are not/lowly mutated and therefore minimise issues with primer annealing. Fifth, cIT-QC rearrangements represent 2/3 of the amplifiable junction classes (in italics in Supplementary Table S1) over all eight primer sets, and thus offer an opportunity to highlight a number of issues, most obviously over-/under-amplification, but also bioinformatic misidentification. Additionally, cIT-QC rearrangements can replace MNC for PCR stability without influencing the patient immune repertoire (since cIT-QC rearrangements are identified and by default excluded from the results).

Our cIT-QC enables the conversion from reads to cells, which is of utmost importance for clinical use. Diagnostic material being analysed for MRD marker identification can show abundances of particular clonotypes that do not reflect the clonal composition of the sample. For example, if the diagnostic sample is highly infiltrated by a lymphoid malignancy that does not harbour a targetable rearrangement, the (few) residual lymphoid cells would generate the whole spectrum of detectable rearrangements; in such situations minor accompanying physiological B or T cell clones could be misassigned as clones with leukaemic markers. In the accompanying study by Brüggemann et al. [21], where 134 clonal signals with abundance >5% were detected by NGS but not by Sanger sequencing, cIT-QC quantification reduced the abundances of 71 (53%) of them below the 5% threshold.

In addition to its use in marker identification, and as exemplarily shown for B and T cell depletion in aplastic follow-up samples, the cIT-QC is of utmost relevance for MRD quantification in samples on or after treatment, in particular if B or T cell-directed therapy was applied, which minimises the background of polyclonal gene rearrangements. If the relative tumour burden is calculated by the ratio of leukaemia-specific reads to all annotated reads without any quantification, the quotient reflects the marker frequency only among cells carrying a particular type of rearrangement (e.g. IG rearrangements in B cells) and might thus heavily overestimate the tumour load [26].

Quantification values over 100% (examples in Fig. 3b and Supplementary Table S5) show that using the cIT-QC is still a semi-quantitative approach, potentially affected by amplification biases. However, there is to date no other scientific or commercial solution available that exceeds our methodology in its broad applicability (universal IG/TR approach) and/or allows precise absolute quantification [12, 27–29].

Finally, the QC protocols are embedded in ARResT/Interrogate, which informs users with reports and messages and allows them, for example, to include the QC-failed samples back into the analysis. The logic behind this is that the ‘fail’ flag simply indicates that our pre-defined QC criteria were not met, and not that the data are corrupt beyond use. Nevertheless, flagged data should always be used with caution, and dependent on the application or question.

In summary, our study showcases the applicability of two reference standards, developed by the EuroClonality-NGS Working Group, which allow standardised analysis of IG/TR NGS data (using the EuroClonality-NGS primer sets) with high reproducibility, accuracy and precision in marker identification. With ARResT/Interrogate, a complete in silico solution accompanying the in vitro assays was built, enabling an analysis of IG/TR sequences including all quality criteria and quantification concepts necessary for valid marker identification in lymphoid malignancies.

## Supplementary information

Supplemental Material

Supp. Table
