# Supplementary material for: Quality control and quantification in IG/TR next-generation sequencing marker identification: protocols and bioinformatic functionalities by EuroClonality-NGS
Source: Leukemia. 2019 Jun 21;33(9):2254–65. doi: 10.1038/s41375-019-0499-4 (PMC6756032; doi:10.1038/s41375-019-0499-4)

**Supplementary Information**

**Quality control and quantification in IG/TR next-generation sequencing marker identification: protocols and bioinformatic functionalities by EuroClonality-NGS**

Henrik Knecht^1^, Tomas Reigl^2^, Michaela Kotrová^1^, Franziska Appelt^1^, Peter Stewart^3^, Vojtech Bystry^2^, Adam Krejci^2^, Andrea Grioni^4^, Karol Pal^2^, Kamila Stranska^2,5^, Karla Plevova^2,5^, Jos Rijntjes^6^, Simona Songia^4^, Michael Svatoň^7^, Eva Froňková^7^, Jack Bartram^8^, Blanca Scheijen^6^, Dietrich Herrmann^1^, Ramón García-Sanz^9^, Jeremy Hancock^10^, John Moppett^11^, Jacques JM van Dongen^12^, Giovanni Cazzaniga^4^, Frédéric Davi^13^, Patricia JTA Groenen^6^, Michael Hummel^14^, Elizabeth A. Macintyre^15^, Kostas Stamatopoulos^16^, Jan Trka^7^, Anton W. Langerak^17*^, David Gonzalez^3^, Christiane Pott^1^, Monika Brüggemann^1^, Nikos Darzentas^1,2^, on behalf of the EuroClonality-NGS Working Group

**Materials and Methods**

**Origin of the healthy thymus, tonsil and MNC used for cPT-QC**

The origin of the various types of materials in the cPT-QC is as follows: healthy human thymus is derived from children undergoing cardiac surgery, from whom the thymus is removed and used upon informed consent as leftover material; tonsil material is also mostly derived from children undergoing surgery for reactive tonsillitis, and is used as leftover material upon informed consent; finally, MNC material is obtained from blood bank donors and used for research purposes upon informed consent.

**Verification of cell line-specific gene rearrangements from human B and T cell lines via ddPCR**

Allele-specific primers for clonal IG/TR rearrangements and probes for quantification were synthesized by Sigma Aldrich. All primers were cleaned by desalting, while hydrolysis probes containing a 5'-FAM/3'-TAMRA reporter dye were cleaned by HPLC. All oligonucleotides were resuspended in TE- buffers (10 mM Tris, pH 8.0, 0.1 mM EDTA) at a total strand concentration Ct = 100 μM and stored at -20°C before use.

ddPCR reactions were prepared in a volume of 20 μL using 10 μL by 2X ddPCR SuperMix (Bio-Rad Laboratories, Hercules, CA), testing two different amounts of cell line gDNA (50ng/500ng) quantified before with the Qubit dsDNA High Sensitivity Assay Kit (Thermo Fisher Scientific, Waltham, MA), forward primer (FP) and reverse primer (RP), each at a final concentration 300 nmol/L, and FAM-labelled probes (100 nmol/L). Droplets were generated by the QX200 droplet generator (Bio-Rad) using 20 μL of the reaction mixture and 70 μL of the droplet generation oil for probes (Bio-Rad), located onto suitable holes in a DG8 cartridge (Bio-Rad). About 45 μL of the drop-oil mixture (12 000 - 20 000 drops) were transferred to a 96-well plate (Bio-Rad) and loaded on a DNA Engine Dyad Peltier Thermal Cycler with the following amplification protocol: 95°C for 10 min, followed by 40 cycles: denaturation at 94°C for 30 s; annealing at 60°C for 1 min; extension at 60°C for 1 min. PCR products were loaded into the QX200 droplet reader and analysed by QuantaSoft Version 1.2 (Bio-Rad Laboratories).

**Mixture preparation of cIT-QC**

Initially, quantification of DNA of selected B and T cell lines was done by Qubit dsDNA High Sensitivity Assay Kit (Thermo Fisher Scientific, Waltham, MA). Quantitative values were checked again by ddPCR-based quantification of the albumin housekeeping gene using 50-200 ng DNA/cell line in order to precisely determine the number of cells per µl of DNA. Primers and probe for albumin quantitation were synthesized by Sigma Aldrich. The sequences of the primers and probe can be found in Supplementary Table S6. ddPCR was carried out according to the protocol described above**,** in duplicates for each cell line. After completion of the PCR, samples were analyzed in the Droplet Reader in terms of number of copies of cell lines per 20µl reaction volume. Based on the values from the ddPCR, the cell line DNA was diluted in TE-buffer down to 400 copies/µl. Thereafter, another ddPCR quantification was performed to check the dilution of each cell line DNA again. Two different volumes of the diluted cell line solution (0.5µl DNA [200 copies] and 2µl DNA [800 copies]) were used as input amount. With suitable quantitative values, cell line DNA’s were further diluted and mixed with each other leading to 40 copies of each cell line being present in 2µl of the DNA mixture. This mixture was added to each sample as cIT-QC and subjected to simultaneous library preparation prior to sequencing.

**Bioinformatics**

Primers

ARResT/Interrogate allows to identify, trim and report on primer sequences, including making the results available for a fully interactive analysis. Primer trimming allows for less artificial sequence data to be processed more accurately and efficiently, and allows for the primer-based results to be used for quality control as described below. Trimming can be controlled even at a per-primer basis: the primer can only be identified and the read left as is; the primer can be kept but the irrelevant read sequence trimmed away; and the primer itself can also be trimmed away. Although primer sequences are artificial and may compromise downstream analyses, it is sometimes necessary (e.g. in highly trimmed junctions) to keep them on to have enough sequence to annotate and thus identify a rearrangement.

Junctions, anchors and junction classes

ARResT/Interrogate is able to annotate and identify different types of rearrangements across all IG/TR loci. We call these rearrangement types ‘junction classes’, listed in full in Supplementary Table S1 per locus and primer set able to amplify them, and shown elsewhere with spike-ins as examples. They include complete, e.g. IG’s VJ:Vh-(Dh)-Jh; incomplete, e.g. TR’s DJ:Db-Jb; and other e.g. IG’s Vk-Kde or intron-Kde. We also list junction classes that are currently only recovered with EuroClonality-NGS capture-based approaches (manuscript in preparation).

For junction classes with no biologically-relevant junctional anchors (i.e. residues that define the CDR3 region, as per IMGT), we decided to introduce virtual ones – this enables consistent and informative results across all junction classes, assisting the user to focus on the most variable part of the rearrangement. For the D genes in DJ, VD and DD incomplete/other junction classes, we use recombination signal sequence (RSS) heptamers (e.g. http://www.imgt.org/IMGTrepertoire/LocusGenes/RecombinationSignals/Hu_index.html): the last triplet of the heptamer in 5’, and the first triplet of the heptamer in 3’. For the intron, we use a CCC triplet between the primer and the RSS heptamer, while for Kde the final triplet after the RSS heptamer and before the primer. In the majority of cases, these anchors are far enough from the junctional point to accommodate more extensive nucleotide trimming, but ARResT/Interrogate is anyway able to use alternative anchors for TRB and TRD, and report rearrangements even with the anchors trimmed or mutated (naturally or artificially) – applicable also to normal anchors in complete rearrangements.

Clonotypes

The different EuroClonality-NGS Working Group activities and the nature of the underlying data led us to develop the following clonotype definition: junction class (rearrangement type), 5’ gene, junctional segmentation and N-(D)-N region statistics (5’ gene deletions, N-(D)-N length, 3’ gene deletions), 3’ gene, junction amino acid sequence, and rearrangement productivity. The segmentation statistics and the junction sequence are based on a strict junction nucleotide sequence clustering that accepts a very limited number of low-abundant sequence differences. Importantly, the user is still able to retrieve and study the original underlying sequence variability.

***
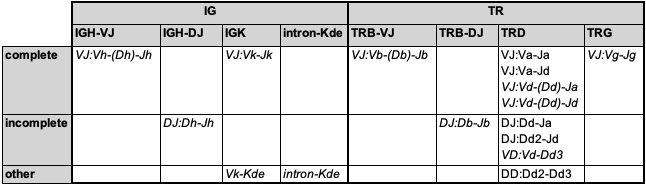
***

**Supplementary Table S1.** Junction classes (representing rearrangement types) produced by the EuroClonality NGS assays and supported by ARResT/Interrogate.


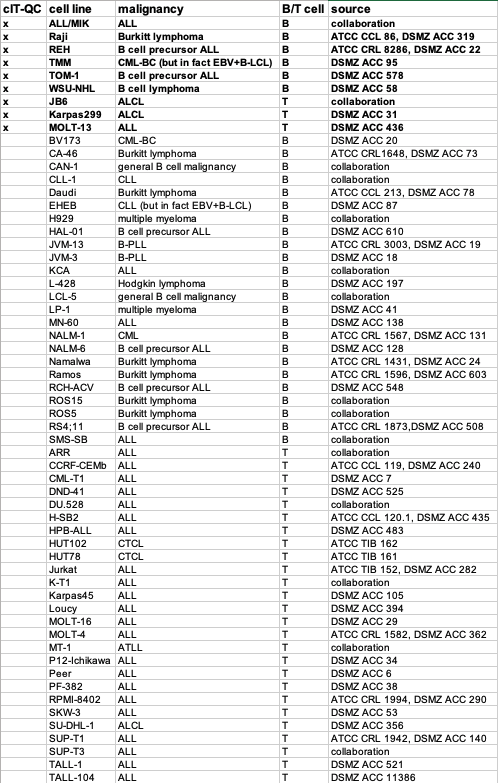


**Supplementary Table S2.** cIT-QC: overview of the human B / T cell lines used to develop the cIT-QC.

**
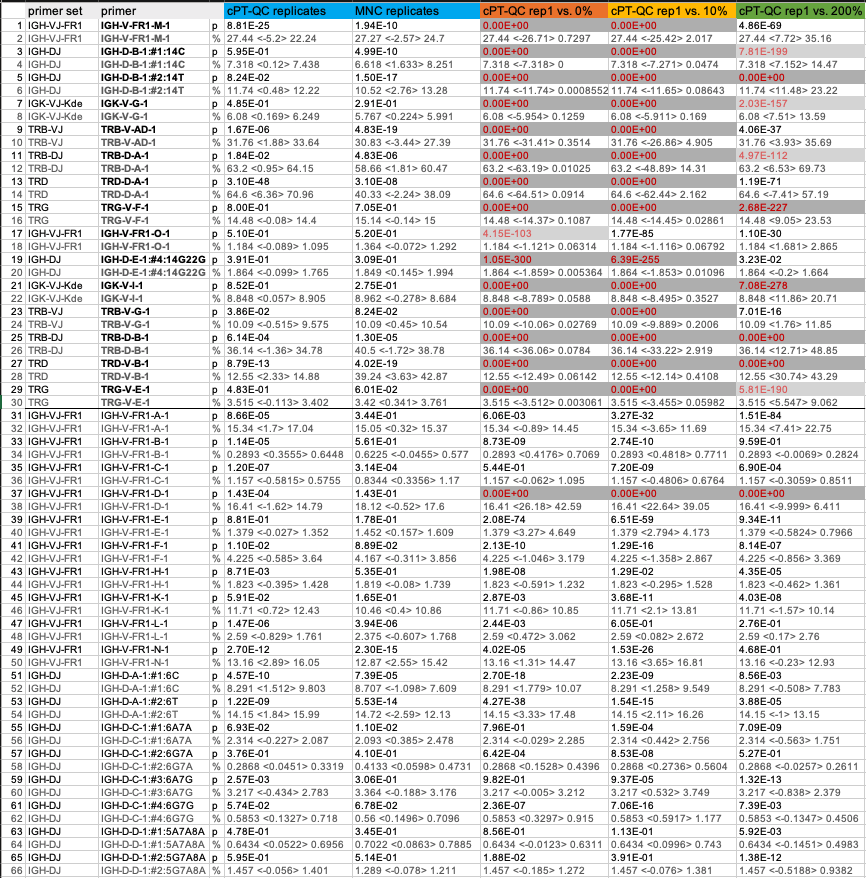

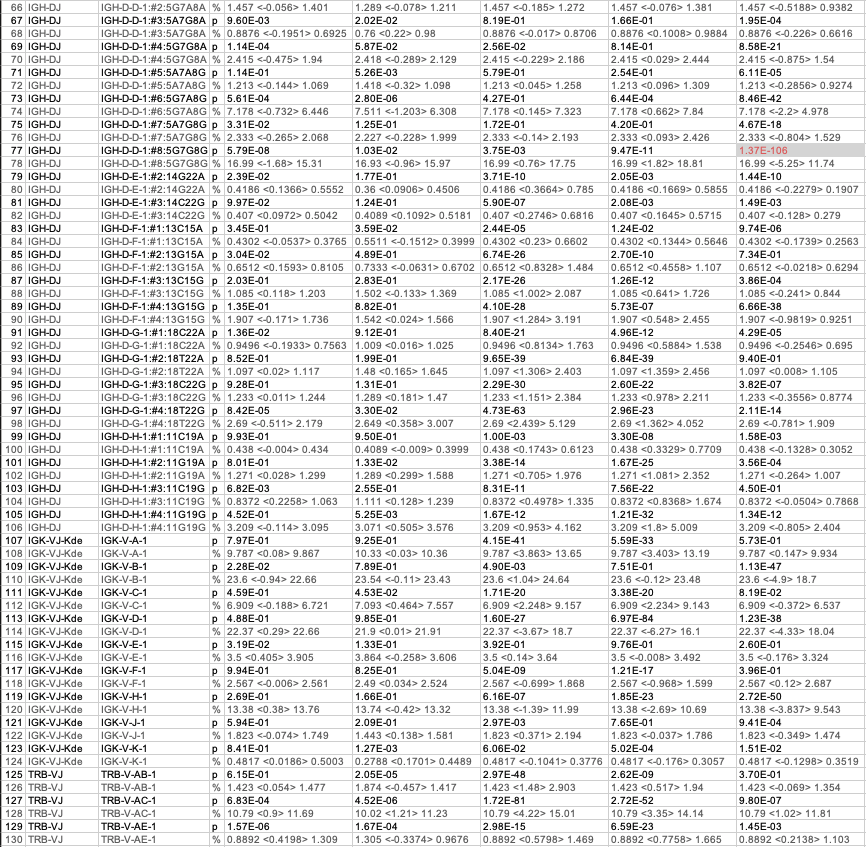

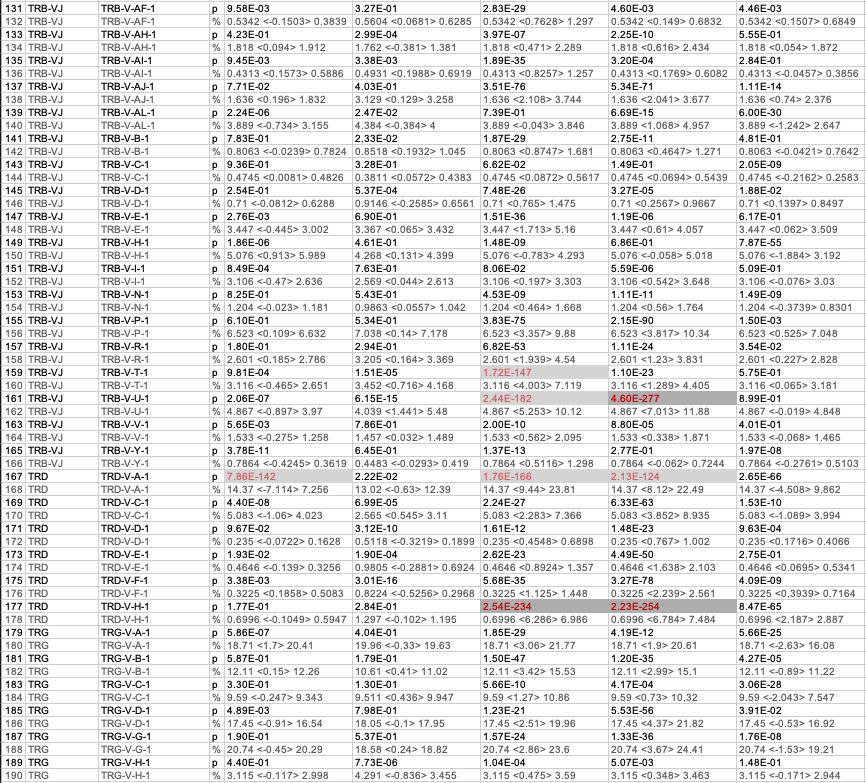
**

**Supplementary Table S3.** cPT-QC: detailed results on replicates and primer perturbations. Compared to Table 1, MNC replicates are included, 5’ primers with at least 10 000 reads across all studied samples (data not shown) are included, p-values are shown and highlighted in grey if below our QC threshold of 1e^-200^.


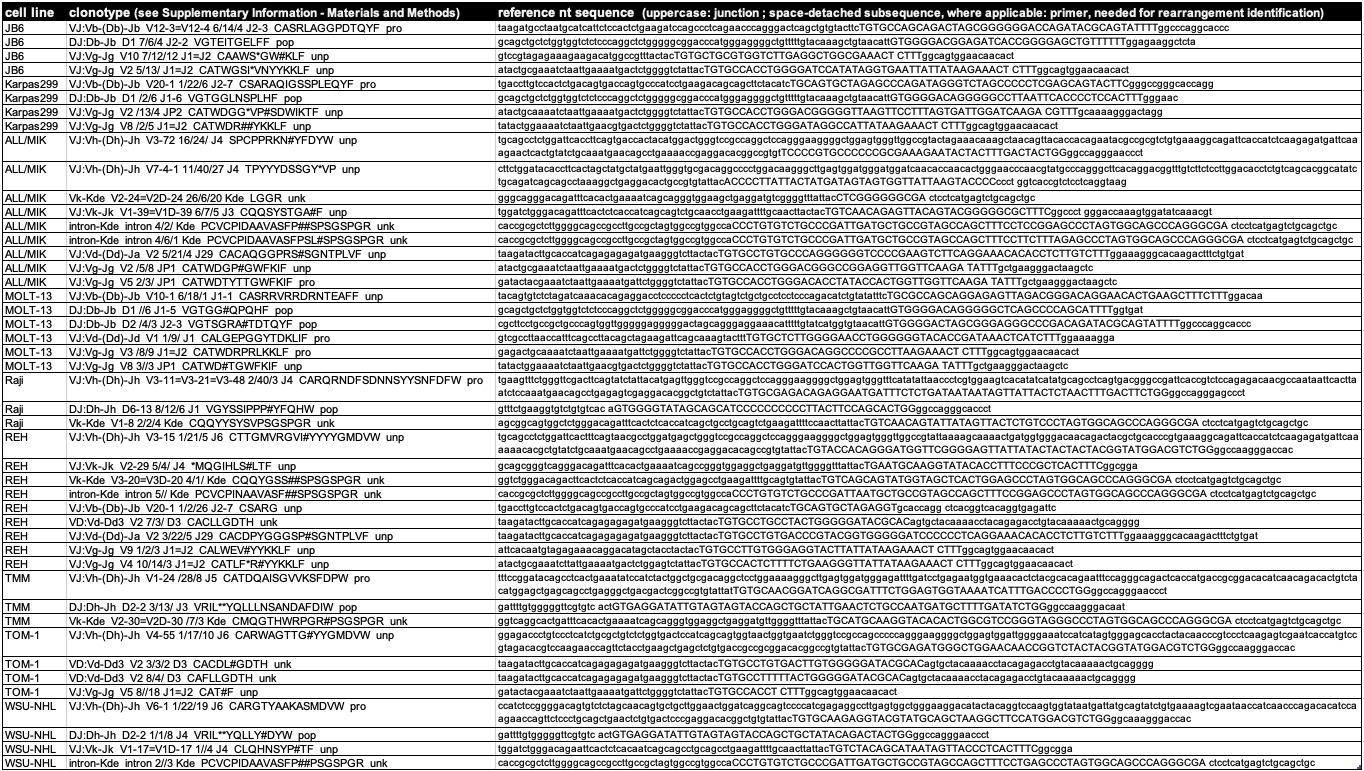


**Supplementary Table S4.** cIT-QC: Table 2 with the reference nucleotide sequences, obtained with the EuroClonality NGS assays as described in the accompanying manuscript by Brüggemann *et al.*, in press. Some columns from Table 2 are omitted for compactness.

**
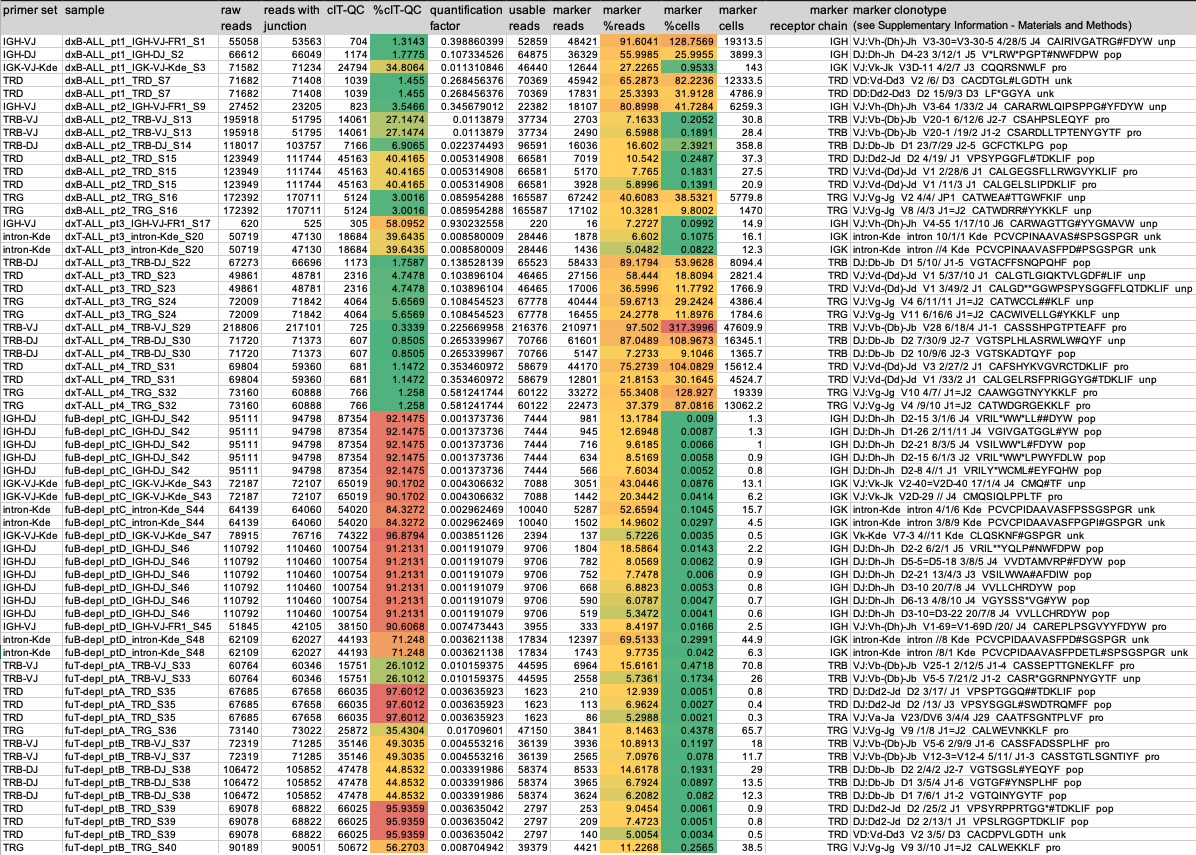
**

**Supplementary Table S5.** Test dataset: markers with >=5% reads before cIT-QC quantification.

| ddPCR_Alb_FP | 5´ CTGGAAGTCGATGAAACATACGTT 3` |
| --- | --- |
| ddPCR_Alb_RP | 5´ CTCTCCTTCTCAGAAAGTGTGCATA 3` |
| ddPCR_Alb_probe | 5´-(6-FAM)-TGCTGAAACATTCACCTTCCATGCAGA-(TAMRA) 3` |

**Supplementary Table S6.** Primers and probe for albumin ddPCR.

**Supplementary Figure S1.** QC-relevant columns of the full ARResT/Interrogate run report produced from the test dataset. The cIT-QC was also sometimes referred to as ‘spikes’ or ‘spike-ins’ in output.


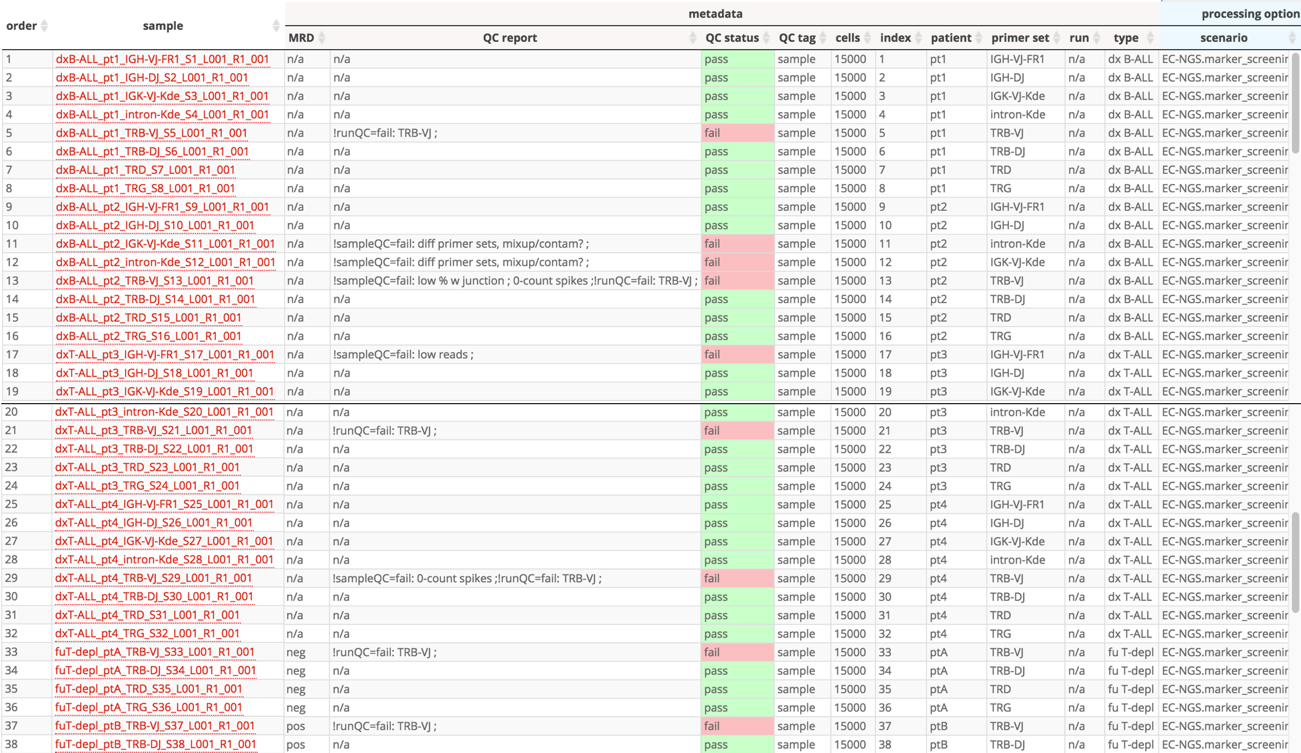

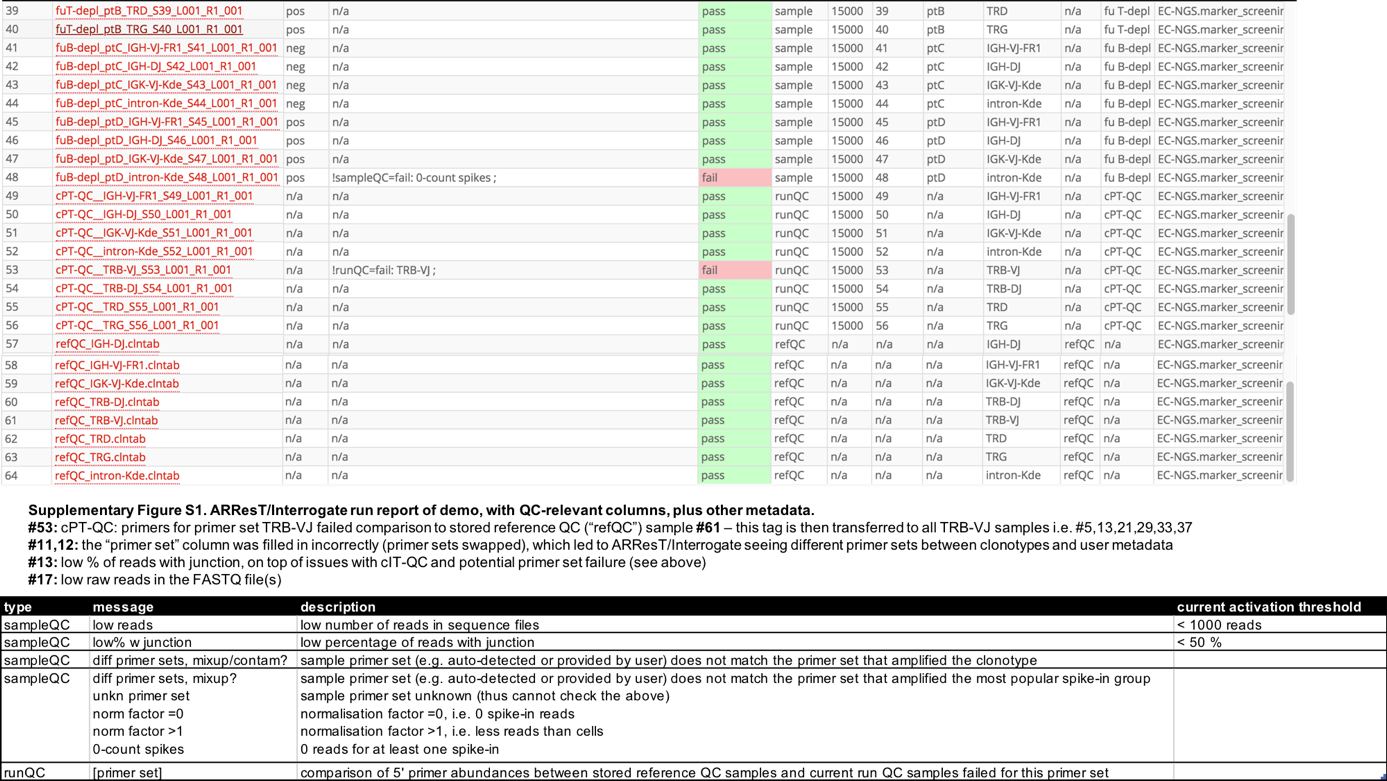

Supplement: Supplementary file 1 — Supplemental Material [file 41375_2019_499_MOESM1_ESM.docx]
